# Cognitive Changes in Older Adults Following a Stepped Care Intervention for Late-life Depression

**DOI:** 10.1093/geroni/igab046.3105

**Published:** 2021-12-17

**Authors:** Anna Y Zhang, Shiyu Lu, Tianyin Liu, Dara K Y Leung, Gloria H Y Wong, Terry Y S Lum

**Affiliations:** 1 The University of Hong Kong, The University of Hong Kong, Not Applicable, Hong Kong; 2 The University of Hong Kong, Hong Kong, Not Applicable, Hong Kong

## Abstract

Older adults with depression may manifest cognitive decline and treating depression may maintain or improve cognition. However, cognitive outcomes could be overlooked in non-pharmacological interventions for depression. This analysis investigated cognitive changes in a stepped-care intervention (Clinical Trial ID: NCT03593889) and the potential association with individual depressive symptom change. The community-dwelling older adults at risk of or with depressive symptoms without significant cognitive impairment (n=802) were assigned to intervention group (n=644) and control group (n=138). Depressive symptoms and cognitive functions were measured using Patient Health Questionnaire-9 and Cognitive Montreal Assessment-5 minutes protocol, respectively. Paired-t-Test showed significant improvements in overall cognition and attention in both intervention and control groups, but the improvements of language fluency (Intervention: MD=-0.51 p<0.01; control: MD=0.14, p=0.500) and orientation (Intervention: MD=-0.22 p<0.05; control: MD=-0.11, p=0.229) only displayed in intervention group. As control group had better cognition at baseline, linear mixed-effects model analysis was used to compare between-group difference. Intervention group had no significant cognitive improvement after adjusting the covariates but a potential improvement in language fluency (Coef. =0.442, SE=0.247, p=0.074). A linear regression analysis in intervention group indicated that reduction of concentration problem (β=0.106, p<0.05) and retardedness (β=0.117, p<0.01) under the symptomatology of depression were associated with the improvement of language fluency. In this group of older persons without significant cognitive impairment, there is no clear evidence of global cognitive benefits in a stepped care depression intervention, although there may be improvements in certain cognitive domains, which may be related to improvements in cognitive aspects of depression.

